# Only a Minority of the Inhibitory Inputs to Cerebellar Golgi Cells Originates from Local GABAergic Cells^1^,^2^,^3^

**DOI:** 10.1523/ENEURO.0055-16.2016

**Published:** 2016-05-23

**Authors:** Mark D. Eyre, Zoltan Nusser

**Affiliations:** Laboratory of Cellular Neurophysiology, Institute of Experimental Medicine, Hungarian Academy of Sciences, 1083, Budapest, Hungary

**Keywords:** cerebellum, GABA, immunohistochemistry, inhibition, patch clamp, synapses

## Abstract

Cerebellar Golgi cells (GoCs) efficiently control the spiking activity of granule cells through GABA_A_ receptor-mediated tonic and phasic inhibition. Recent experiments provided compelling evidence for the extensive interconnection of GoCs through electrical synapses, but their chemical inhibitory synaptic inputs are debated. Here, we investigated the GABAergic synaptic inputs of GoCs using *in vitro* electrophysiology and quantitative light microscopy (LM) and electron microscopy (EM). We characterized GABA_A_ receptor-mediated IPSCs in GoCs and Lugaro cells (LuCs), and found that IPSCs in GoCs have lower frequencies, smaller amplitudes, and much slower decay kinetics. Pharmacological and LM immunolocalization experiments revealed that GoCs express α3, whereas LuCs express α1 subunit-containing GABA_A_ receptors. The selective expression and clustered distribution of the α3 subunit in GoCs allowed the quantitative analysis of GABAergic synapses on their dendrites in the molecular layer (ML). EM and LM experiments in rats, and wild-type and GlyT2-GFP transgenic mice revealed that only one third of axon terminals establishing GABAergic synapses on GoC dendrites contain GlyT2, ruling out LuCs, globular cells, and any noncortical glycinergic inputs as major inhibitory sources. We also show that axon terminals of stellate/basket cells very rarely innervate GlyT2-GFP-expressing GoCs, indicating that only a minority of the inhibitory inputs to GoCs in the ML originates from local interneurons, and the majority of their inhibitory inputs exclusively releases GABA.

## Significance Statement

Golgi cells are essential for controlling the activity of granule cells in the cerebellum by releasing the inhibitory neurotransmitter GABA, but very little is known about the sources of their own inhibition. We used functional and morphological techniques to demonstrate that the inhibitory postsynaptic receptors on Golgi cells are unique among the cell types in the cerebellar cortex, and used these unique GABA_A_ receptors to visualize Golgi cell inhibitory synapses and their presynaptic inputs. This study extends our understanding of cerebellar microcircuits by demonstrating that only a minority of the inhibitory inputs to Golgi cells originate from GABAergic cells of the cerebellar cortex.

## Introduction

The essential role of Golgi cells (GoCs) in higher cerebellar functions was elegantly demonstrated by Watanabe et al. (1998) by showing severe deficits in motor coordination following their selective pharmacological ablation. To understand the way these GABAergic interneurons (INs) fulfill their roles in circuit dynamics, their intrinsic properties and synaptic inputs and outputs need to be quantitatively determined. GoCs are located in the granule cell layer (GCL) of the cerebellar cortex and comprise a molecularly heterogeneous population of cells (Vincent et al., 1985; Ohishi et al., 1994; Neki et al., 1996; Singec et al., 2003; Simat et al., 2007). Despite this heterogeneity, their classifying morphological features are an axonal arborization restricted to the GCL and a characteristic dendritic arbor that extends into the molecular layer (ML; Eccles et al., 1967; Palay and Chan-Palay, 1974). Although there are additional GABAergic INs in the GCL [Lugaro cells (LuCs) and globular cells], GoCs provide the sole source of GABAergic inhibition to granule cells (GrCs), and efficiently control the spiking activity of GrCs through GABA_A_ receptor (GABA_A_R)-mediated tonic and phasic inhibition (Brickley et al., 1996; Farrant and Nusser, 2005; Silver, 2010). Golgi cells receive feedforward and feedback excitatory inputs from mossy fibers and parallel fibers in the GCL and ML, respectively (Eccles et al., 1967; Dieudonne, 1998; Vos et al., 1999; Kanichay and Silver, 2008). It is also widely accepted that they are richly interconnected through electrical synapses (Dugué et al., 2009; Vervaeke et al., 2010), underlying the synchronization or desynchronization of spontaneously active GoC networks.

Based on the location of GoC dendrites in all layers of the cerebellar cortex, all GABAergic cells that have axonal arbors in the cortex could, in principle, provide GABAergic synaptic inputs to GoCs. Synaptic GABAergic inhibition onto GoCs has historically been assumed to arise from ML INs (MLIs; i.e. stellate and basket cells), based on the electron microscopy (EM) observation of symmetric synapses onto GoC dendrites (Palay and Chan-Palay, 1974) and the presence of IPSCs following electrical stimulation in the ML (Dumoulin et al., 2001). However, a recent study (Hull and Regehr, 2012) using selective light-mediated activation of MLIs expressing channelrhodopsin2 challenged this view by demonstrating a lack of functional connectivity between MLIs and GoCs. Several studies have reported the lack of chemical inhibitory connectivity between GoCs, ruling this out as the major source of their inhibitory inputs (Dieudonne, 1998; Dugué et al., 2009; Vervaeke et al., 2010), although some connectivity among GoCs has been reported (Hull and Regehr, 2012).

Dieudonné and Dumoulin (2000) demonstrated that the application of serotonin (5-HT) evokes action potential (AP)-dependent mixed GABAergic and glycinergic IPSCs in GoCs, which are likely to arise from LuCs. Lugaro cells are GCL INs with horizontal dendritic arbors running parallel with the Purkinje cell layer (PCL) and axons arborizing mainly in the ML and to a lesser extent in the GCL. LuCs have been shown to form synapses onto IN dendrites in the ML (Lainé and Axelrad, 1998), but little is known about their exact postsynaptic target cells or their presynaptic inputs, apart from the presence of calbindin-immunoreactive boutons surrounding their somata that were assumed to be Purkinje cell local axon collaterals (Lainé and Axelrad, 2002; Simat et al., 2007).

Considerable variability has been observed in the morphological features of GCL INs. In addition to GoCs and LuCs, globular and candelabrum cells have also been discerned (Lainé and Axelrad, 1994; Hirono et al., 2012). There is also heterogeneity among GoCs; five subtypes have been identified based on soma size and molecular content [e.g., glycine transporter type 2 (GlyT2), GAD67, mGluR2, or neurogranin; Simat et al., 2007], but the functional consequences of this heterogeneity are unknown. Because LuCs, globular cells, and most GoCs express GlyT2, we have used mice in which enhanced green fluorescent protein (EGFP) is expressed by bacterial artificial chromosome insertion under the control of the GlyT2 promoter (Zeilhofer et al., 2005) as a marker to investigate the inhibitory inputs and outputs of GCL INs.

## Materials and Methods

### Animals

Male and female mice heterozygous for the bacterial artificial chromosome insertion of EGFP under the control of the glycine transporter type 2 gene (Zeilhofer et al., 2005; henceforth GlyT2-GFP mice), wild-type (WT) C57Black6J mice (henceforth WT mice), and Wistar rats were killed by decapitation, in accordance with local laws and with the ethical guidelines of the Institute of Experimental Medicine Protection of Research Subjects Committee.

### Acute slice preparation

GlyT2-GFP mice (*n* = 30; mean age, 26.7 ± 5.2 d) were deeply anesthetized with isoflurane (Abbott Laboratories). After decapitation, the brain was removed and placed into a sucrose-based, ice-cold artificial CSF (ACSF) containing the following (in mm): 230 sucrose, 2.5 KCl, 25 glucose, 1.25 NaH_2_PO_4_, 24 NaHCO_3_, 4 MgCl_2_, and 0.5 CaCl_2_, bubbled continuously with 95% O_2_ and 5% CO_2_, resulting in a pH of 7.4. In order to investigate the factors contributing to the spontaneous activity of GoCs, we conducted experiments with two male Wistar rats (ages, 20 and 21 d) using the above solution, and also two GlyT2-GFP mice (ages: male, 25 d; female, age 26 d), two male WT mice (ages, 21 and 24 d), and three male Wistar rats (ages, 18, 19, and 24 d) using a different, K-gluconate-based, ice-cold cutting solution. This solution contained the following (in mm): 130 K-gluconate, 15 KCl, 0.05 EGTA, 20 HEPES, 25 glucose, and 3 kynurenic acid, pH adjusted to 7.4 with NaOH. In all cases, parasagittal slices from the cerebellar vermis were cut at a thickness of 250 μm with a Vibratome (VT1000S; Leica) and were stored in ACSF containing the following (in mm): 126 NaCl, 2.5 KCl, 25 glucose, 1.25 NaH_2_PO_4_, 24 NaHCO_3_, 2 MgCl_2_, and 2 CaCl_2_, bubbled continuously with 95% O_2_ and 5% CO_2_, resulting in a pH of 7.4. After a 30 min recovery period at 33°C, slices were further incubated at room temperature until they were transferred to the recording chamber.

### Electrophysiological recordings

Somatic whole-cell recordings were performed at 26.7 ± 0.9°C using infrared differential interference contrast on an Olympus BX51WI Microscope with a 40× water-immersion objective. All voltage- and current-clamp recordings were performed using a mixed K-gluconate- and KCl-based intracellular solution containing the following (in mm): 65 K-gluconate, 70 KCl, 2.5 NaCl, 1.5 MgCl_2_, 0.025 EGTA, 10 HEPES, 2 Mg-ATP, 0.4 Mg-GTP, 10 creatinine phosphate, and 8 biocytin, pH 7.33 and 270–290 mOsm. The reversal potential for chloride ions was calculated as −15.3 mV (http://www.physiologyweb.com/calculators/nernst_potential_calculator.html). All recordings were performed in ACSF in the presence of 3 mm kynurenic acid to inhibit ionotropic glutamate receptors. We investigated the spontaneous neuronal activity of GoCs recorded from Wistar rats and WT mice prepared using both cutting solutions, and in GlyT2-GFP mouse slices cut in the K-gluconate-based solution, for 3 min in cell-attached mode prior to attaining the whole-cell configuration. A series of constant hyperpolarizing and depolarizing current pulses with incremental amplitudes was applied to each cell in order to elicit voltage responses and suprathreshold action potential firing patterns. Continuous DC currents were not applied to cells to maintain them at a specified membrane potential. For voltage-clamp recordings of miniature IPSCs (mIPSCs) at a holding potential of −70 mV, 1 μm tetrodotoxin (TTX; Alomone Labs) was either included in the ACSF or washed into the bath. After establishing the whole-cell configuration and allowing for a 2 min stabilization period, a period of 4 min was recorded for each cell (“baseline”). For pharmacological experiments, the perfusion solution was changed to one containing ACSF, 100 nm 2',4-difluoro-5'-[8-fluoro-7-(1-hydroxy-1-methylethyl)imidazo[1,2-*a*]-pyridin-3-yl]-[1,1'-biphenyl]-2-carbonitrile (TP003; Tocris Bioscience) or 100 nm zolpidem (Sigma-Aldrich). After a 12 min period of drug equilibration, a second 4 min period (“steady state”) was recorded. In a subset of recordings, the drug solution was then changed to one containing the drug plus SR95531 (20 μm; Sigma-Aldrich). In some experiments, spontaneous IPSCs (sIPSCs) were recorded for 1 min after establishing the whole-cell configuration, and then TTX was washed into the bath. All recordings were performed with MultiClamp 700A and 700B amplifiers (Molecular Devices). Patch pipettes were pulled (Universal Puller; Zeitz-Instrumente Vertriebs) from thick-walled borosilicate glass capillaries with an inner filament (1.5 mm outer diameter, 0.86 mm inner diameter; Sutter Instruments). Data were digitized on-line at 20 kHz and filtered at 3 kHz with a low-pass Bessel filter. For voltage recordings, resting membrane potential (RMP) values [steady-state responses used to calculate the input resistance and the Sag ratio (the ratio of peak vs steady state of the hyperpolarizing voltage response)] uncorrected for liquid junction potential were measured manually; action potential properties (e.g., threshold, amplitude) were detected using a custom-made software written in Python [AP threshold was defined as the voltage at which the first derivative of the voltage trace reaches 10% of its peak; AP amplitude was defined as the difference between the AP threshold voltage and the most depolarized voltage of the AP; afterhyperpolarization (AHP) amplitude was defined as the difference between the AP threshold voltage and the most hyperpolarized voltage of the AP]. For current recordings, individual mIPSCs were detected as inward current changes above a variable threshold for 1.2 ms, referenced to a 2.5 ms baseline period, and analyzed off-line using EVAN 1.5 (Nusser et al., 2001). The detection thresholds were similar between cell types (Golgi cells: 1.78 ± 0.23 pA; range, 0.5–3.0 pA; Lugaro cells: 2.14 ± 0.24 pA; range, 1.5–3.0 pA). Traces containing overlapping synaptic currents in their decaying phase were discarded from the analysis of decay times. Access resistance (Ra) was subject to 70% compensation and was continuously monitored. If Ra changed >20% during the recording, the cell was discarded from the analysis. All recordings were rejected if the uncompensated Ra became >20 MΩ. After recordings, slices were fixed in 0.1 m phosphate buffer (PB) containing 2% paraformaldehyde (PFA; Molar Chemicals) and 15% v/v picric acid (PA) for 24 h before *post hoc* visualization of the biocytin-filled cells.

### *Post hoc* visualization of biocytin-filled cells

Slices were washed several times in 0.1 m PB, embedded in agar, and resectioned at 60 μm thickness with a Vibratome. Sections were then washed in Tris-buffered saline (TBS), blocked in TBS containing 10% normal goat serum (NGS) for 1 h, and then incubated in TBS containing rabbit anti-GFP (1:1000; Millipore), 2% NGS, and 0.1% Triton X-100 overnight at 24°C. Sections were then washed three times in TBS, incubated in TBS containing Alexa Fluor 488-conjugated goat anti-rabbit IgG (1:500; Life Technologies), Cy3-conjugated streptavidin (1:500; Jackson ImmunoResearch), 2% NGS, and 0.1% Triton X-100 for 2 h, followed by washing and mounting on glass slides in Vectashield (Vector Laboratories). In some cases, other primary antisera were used [mouse anti-GFP (1:1000; NeuroMab) and rabbit anti-calbindin IgGs (1:1000; Swant)], and the secondary antisera used were Alexa Fluor 488-conjugated goat anti-mouse (1:500; Life Technologies) and Cy5-conjugated goat anti-rabbit (1:500; Jackson ImmunoResearch) IgGs. Images were acquired using a confocal laser-scanning microscope (FV1000; Olympus) with a 20× [numerical aperture (NA), 0.7) or a 60× (NA, 1.35)] objective. *Z*-stack images were acquired for cell identification, morphological reconstruction, and quantification with the Neurolucida System (MBF Bioscience). The axons of MLIs were fully reconstructed from the point where they emerged from the soma, and markers were placed at axonal varicosities.

### Immunofluorescent reactions and quantification

Male adult (*n* = 4; age, 70 d; for neurogranin immunolabeling) or juvenile (*n* = 2; age, 21 and 28 d; for GlyT2 immunolabeling) GlyT2-GFP mice, three male adult WT mice (age, 35, 35, and 68 d), or two male adult Wistar rats (both age 42 d; for GlyT2 immunolabeling) were anesthetized initially with isoflurane followed by ketamine (www.vetcentre.com; 0.5 ml/100 g body weight, i.p.), and were then transcardially perfused with 0.9% saline for 2 min followed by 2% PFA in 0.1 m sodium acetate buffer, pH 6.0, for 15 min. The cerebella were dissected and then washed three times in PB. Vibratome sections were cut at 60 μm. All sections were then washed in TBS, followed by blocking in TBS containing 10% NGS for 1 h. The sections were then incubated in a solution containing single or mixtures of primary antisera made up in TBS containing 0.1% Triton X-100 and 2% NGS overnight at 24°C. Next, sections were incubated for 2 h in appropriate secondary antisera made up in TBS containing 2% NGS, then were washed and mounted in Vectashield (Vector Laboratories). The following primary and secondary antisera were used for the experiments illustrated in Figure 3, *A* and *B*: rabbit anti-GABA_A_R α3 (1:1000; Synaptic Systems) and Alexa Fluor 488-conjugated goat anti-rabbit (1:500; Invitrogen/Molecular Probes); guinea pig anti-neuroligin-2 (1:500; Frontier Institute) and Cy3-conjugated donkey anti-guinea pig (1:500; Jackson ImmunoResearch); and mouse anti-GABA_A_R β3 (1:1000; NeuroMab) and Cy5-conjugated goat anti-mouse (1:1000; Jackson ImmunoResearch). The following primary and secondary antisera were used for the experiments illustrated in Figure 3*C–E*: mouse anti-GFP (1:1000; NeuroMab) and Alexa Fluor 488-conjugated goat anti-mouse (1:500; Invitrogen/Molecular Probes); guinea pig anti-GABA_A_R α3 subunit (1:500; Synaptic Systems) and Cy3-conjugated donkey anti-guinea pig (1:500; Jackson ImmunoResearch); and rabbit anti-neurogranin (1:1000; Millipore) and Cy5-conjugated goat anti-rabbit (1:1000; Jackson ImmunoResearch). The following primary and secondary antisera were used for the experiments illustrated in Figure 5: guinea pig anti-GlyT2 (1:5000; Millipore) and Cy3-conjugated donkey anti-guinea pig (1:500; Jackson ImmunoResearch); rabbit anti-GABA_A_R α3 subunit (1:1000; from W. Sieghart, Medical University of Vienna, Austria) and Cy5-conjugated goat anti-rabbit (1:500; Jackson ImmunoResearch). *Z*-stack images were acquired in a random manner using a confocal laser-scanning microscope (FV1000) with a 20× or a 60× objective. Golgi cell dendrites were reconstructed, and GABA_A_R α3-immunoreactive puncta were indicated by markers using the Neurolucida software. Two adjacent edges and the upper focal plane were used as exclusion boundaries for stereological optical disector counting of markers. The number of markers divided by the volume of the confocal stack was used to calculate synapse density estimates.

### Electron microscopy

Female adult GlyT2-GFP mice (*n* = 5; age, 38.4 ± 10.5 d) were anesthetized initially with isoflurane inhalation, followed by ketamine, and then transcardially perfused with 0.9% saline for 2 min followed by a fixative containing 4% PFA and 15% v/v PA in 0.1 m PB with 0%, 0.01%, or 0.1% glutaraldehyde for 15–30 min. The cerebella were then washed three times in PB, and Vibratome sections were cut at 70 μm. Sections were cryoprotected in 10% sucrose in PB for 1 h and then 30% sucrose overnight at 4°C, followed by freezing in liquid nitrogen and thawing in PB. Sections were then treated with 1% H_2_O_2_ in PB for 10 min, incubated with mouse anti-GFP IgG (1:500; NeuroMab) diluted in TBS with 2% NGS overnight at 24°C, followed by biotinylated goat anti-mouse (1:50; Vector Laboratories) antibody diluted 1:50 in TBS with 2% NGS for 2 h, and then an avidin–biotin complex (Vector Laboratories) diluted in TBS overnight at 24°C, and then with diaminobenzidine (DAB; 0.05% solution in Tris buffer, pH 7.4) as chromogen and 0.01% H_2_O_2_ as oxidant for 2 min. Sections were then postfixed in 1% OsO_4_ for 20 min, stained with 1% uranyl acetate for 25 min, dehydrated in a graded series of ethanol, and embedded in epoxy resin (Durcupan). Sections were re-embedded, and serial sections were cut at 70 nm thickness using an ultramicrotome (Ultracut; Leica Microsystems) and collected onto copper Pioloform-coated slot grids. Sections were viewed using a JEM-1011 Transmission Electron Microscope (JEOL), and digital images of all labeled profiles present in a section were captured with a cooled CCD camera (Cantega G2 Camera; Olympus Soft Imaging Solutions GmbH).

### Statistical procedures

All data are expressed as the mean ± SD throughout this article. All statistical comparisons were made with Statistica 11 Software (Scientific Computing). Intrinsic electrical properties datasets failing the Shapiro–Wilk normality test were compared using the nonparametric Mann–Whitney *U* test; data passing this test were compared by one-way parametric ANOVA. Comparisons of data pertaining to the spontaneous firing of GoCs in the three different genotypes were made using either a nonparametric Kruskal–Wallis test or a one-way parametric ANOVA, according to the results of the Shapiro–Wilk normality tests. sIPSC and mIPSC properties and GABA_A_R pharmacology data passed the Shapiro–Wilk normality test, and were compared by one-way parametric ANOVA, with ACSF baseline and drug steady-state conditions in the same cells treated as repeated measures (rm-ANOVA). Where appropriate, data were further assessed by conducting a *post hoc* test (Tukey’s unequal *n* HSD *post hoc* test; Tukey’s unequal *n*; or the Kruskal–Wallis multiple-comparisons test). Differences were considered significant at *p* < 0.05.

## Results

**Figure 1.**
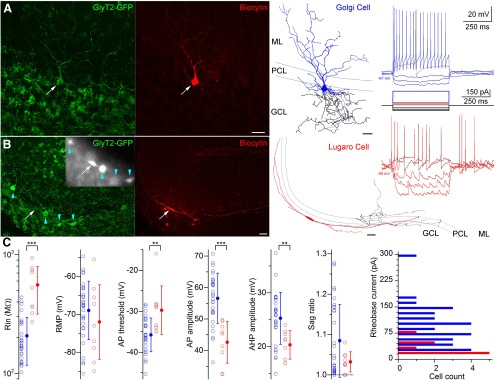
Morphological and electrophysiological characterization of granule cell layer interneurons in the GlyT2-GFP mouse. ***A***, Maximum intensity projection image of a confocal image *Z*-stack of an *in vitro* recorded and biocytin (red)-labeled GoC showing GlyT2-GFP immunoreactivity (green). Scale bar, 20 μm. The Neurolucida reconstruction of this cell is shown on the right (dendrites in blue; axon in black). Scale bar, 20 μm. Voltage responses of this cell to DC current injections are shown on the far right. ***B***, As in ***A***, but showing a Lugaro cell. Although the GFP signal was not detectable in this cell after the recording, prior to patching this cell was clearly GFP positive, as shown in the epifluorescent grayscale image in the inset (arrow; arrowheads indicate adjacent GlyT2-GFP-expressing neurons). The Neurolucida reconstruction of this cell is shown on the right (dendrites in red; axon in black). Scale bar, 20 μm. Voltage responses of this cell to DC current injections are shown on the far right. Note the frequent occurrence of spontaneous IPSPs in the traces. The IPSPs are depolarizing due to the elevated Cl^−^ concentration of the intracellular solution. ***C***, Summary of some electrophysiological properties of morphologically identified Golgi (blue) and Lugaro (red) cells. Rin, Input resistance; Rheobase, minimum current required to generate at least one action potential. **p < 0.01, ***p < 0.001. Testing membrane potential (RMP) and Sag ratio were not different between cell types (*p* = 0.34 and *p* = 0.46, respectively). Average rheobase current was significantly different between cell types (*p* = 0.0002). Comparisons were made by Mann–Whitney *U* test or one-way ANOVA.

Because GFP is expressed in distinct types of GABAergic INs of the GCL in GlyT2-GFP mice (GoCs, LuCs, and globular cells; Zeilhofer et al., 2005; Simat et al., 2007), and their cell type-specific features cannot be easily identified in acute slices due to the high density and extensive overlap among the numerous GFP-containing axonal and dendritic processes, we *post hoc* identified all of our recorded and biocytin-filled INs. We successfully recovered 39 neurons with sufficient amounts of axons and dendrites for unequivocal identification as either GoCs (*n* = 30; Fig. 1*A*) or LuCs (*n* = 9; Fig. 1*B*). GoCs had somata of variable sizes, several basal dendrites, and apical dendrites crossing the PCL, and ramifying to different extents in the ML. Their axons extensively arborized in the GCL. LuCs had horizontally elongated somata and dendrites running parallel with and under the PCL in the GCL, and a sparsely ramifying axon in the ML. The axons of LuCs were frequently truncated. Neither globular cells nor LuCs with somatic locations deep in the GCL were present in our sample.

Differences in the responses of GoCs (*n* = 30) and LuCs (*n* = 9) to hyperpolarizing and depolarizing current injections were readily observed (Fig. 1*A*,*B*). Quantitative comparisons indicated that current rheobase was significantly larger (98.0 ± 58.0 vs 30.0 ± 21.2 pA; *p* = 0.0002, Mann–Whitney *U* test), and input resistance was significantly lower (207.0 ± 89.8 vs 556.3 ± 239.5 MΩ; *p* = 0.0001, Mann–Whitney *U* test; Fig. 1*C*) in GoCs versus LuCs. AP parameters also varied between cell types, as follows: GoCs had a significantly more negative AP threshold (−35.8 ± 4.1 vs −29.7 ± 5.9 mV; *p* = 0.0041, Mann–Whitney *U* test), larger AP amplitude (56.6 ± 8.0 vs 42.6 ± 6.6 mV; *p* = 0.00003, one-way ANOVA), and larger afterhyperpolarization amplitude (25.3 ± 4.8 vs 20.3 ± 2.7 mV; *p* = 0.0059, one-way ANOVA; Fig. 1*C*) than LuCs. In contrast, the resting membrane potential (−69.0 ± 7.7 vs −72.4 ± 9.1 mV; *p* = 0.34, one-way ANOVA), and Sag ratio (1.08 ± 0.09 vs 1.03 ± 0.03; *p* = 0.46, Mann–Whitney *U* test; Fig. 1*C*) were not significantly different between GoCs and LuCs. However, by far the most reliable difference between the two cell types was the much higher frequency of spontaneous synaptic potentials in LuCs (Fig. 1*A*,*B*; see also Hirono et al., 2012).

We therefore recorded sIPSCs and mIPSCs from a subset of these GoCs (*n* = 12) and LuCs (*n* = 7) in the whole-cell voltage-clamp mode. All data passed the Shapiro–Wilk test and were compared with an rm-ANOVA followed by Tukey’s unequal *n* test when appropriate. Spontaneous IPSCs were significantly less frequent (0.4 ± 0.2 vs 10.9 ± 5.0 Hz; *p* = 0.0002), were significantly smaller in amplitude (10.5 ± 3.1 vs 21.1 ± 7.2 pA; *p* = 0.0033), and had a significantly larger mean weighted decay time constant (τ_w_; 20.3 ± 10.1 vs 5.5 ± 0.6 ms; *p* = 0.0042) in GoCs compared with LuCs, which is consistent with our qualitative assessment based on current-clamp recordings. The application of TTX had a minimal effect on the frequency of IPSCs in GoCs (reduced to 0.3 ± 0.2 Hz; *n* = 12, *p* = 1.00), but significantly reduced the frequency in LuCs (reduced to 6.6 ± 3.1 Hz; *n* = 7, *p* = 0.0026). In contrast, neither IPSC peak amplitude (10.3 ± 2.7 pA for GoCs; 21.7 ± 7.2 pA for LuCs; *p* = 0.52, rm-ANOVA) nor τ_w_ (21.6 ± 7.7 ms for GoCs; 5.5 ± 0.6 ms for LuCs; *p* = 0.69, rm-ANOVA) were significantly affected for either cell type. The lack of TTX effect on the frequency of IPSCs in GoCs suggests that a large fraction of cell bodies providing the inhibitory inputs to GoCs was either spontaneously silent or was present outside of our *in vitro* slice. In contrast, LuCs receive inhibitory inputs from at least one spontaneously active local source, which is consistent with the results of the study by Hirono et al. (2012).

Several studies have reported the presence or absence of spontaneous spiking activity of GoCs in acute slices (Dieudonne, 1998; Dugué et al., 2005 , 2009; Forti et al., 2006; Vervaeke et al 2010; Hirono et al., 2012; Rudolph et al., 2015) and have suggested that the lack of spontaneous activity might reflect deficiencies in the quality of the slices (Rudolph et al., 2015). In our initial dataset in GlyT2-GFP mice, we observed that only 7% of GoCs (2 of 30) displayed spontaneous activity, whereas spontaneous firing was not observed in LuCs. We investigated GoC spontaneous spiking activity in more detail by using two different cutting solutions (sucrose-based or K-gluconate-based), and compared GlyT2-GFP mouse, WT mouse (C57Black6J), and Wistar rat GoCs by measuring the spontaneous firing frequencies in the cell-attached configuration for 3 min prior to attaining the whole-cell configuration. We found that the occurrence of spontaneous activity was low in GoCs from GlyT2-GFP (21%; 6 of 28 cells) and WT (13%; 1 of 8 cells) mouse slices cut in K-gluconate (median and maximum frequencies in GlyT2-GFP: 0 and 0.58 Hz; in WT mice: 0 and 2.16 Hz). In contrast, Wistar rat GoCs frequently exhibited spontaneous activity, either using sucrose-based (69%; 9 of 13 cells) or K-gluconate-based (79%; 11 of 14 cells) cutting solutions (median and maximum frequencies: sucrose, 2.9 and 9.9 Hz; gluconate, 3.2 and 11.3 Hz). When we quantitatively analyzed the data from slices prepared with the K-gluconate solution across genotypes, the mean spontaneous frequency of rat GoCs (3.4 ± 3.1 Hz, *n* = 14) was significantly higher than that found in both GlyT2-GFP and WT mouse GoCs (0.1 ± 0.2 Hz, *n* = 15, *p* = 0.0048; and 0.3 ± 0.8 Hz, *n* = 8, *p* = 0.0023, respectively, Kruskal–Wallis multiple comparisons *post hoc* test).

We also measured the frequency, peak amplitude, and kinetic parameters of sIPSCs, and found no significant differences between genotypes for any of these measured parameters (e.g., sIPSC τ_w_; rat, 21.0 ± 11.1 ms; GlyT2-GFP mouse, 24.4 ± 10.0 ms; WT mouse, 27.6 ± 9.3 ms; *p* = 0.15, one-way ANOVA), which were very similar to those found in GlyT2-GFP mouse GoCs prepared using the sucrose-based cutting solution (see above). All of these data taken together indicate species-specific variability in the spontaneous activity of GoCs in acute slices, but our data do not rule out any additional mechanism. Because the τ_w_ of sIPSCs in GoCs recorded from GlyT2-GFP mice, WT mice, and rats were very similar, we performed most of the following experiments in GlyT2-GFP mice.

**Figure 2.**
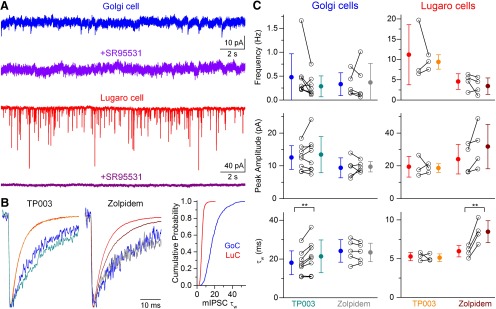
GoCs and LuCs have distinct mIPSC properties and different GABA_A_R α subunit content. ***A***, Continuous current recordings from a GoC (blue) and a LuC (red) in the presence of 1 μm tetrodotoxin before and after the application of 20 μm SR95531 (traces below). Note the greater frequency and larger amplitude of mIPSCs in the LuC, and that all currents were eliminated by the GABA_A_R antagonist. ***B***, Average, peak-scaled traces from example cells before (GoC, blue; LuC, red) and after bath application of 100 nm TP003 (left: GoC, teal; LuC, orange) or 100 nm zolpidem (middle: GoC, gray; LuC, dark red). Note the much faster mIPSC decay and selective prolongation by 100 nm zolpidem in the LuC, compared with the slower decay and selective prolongation by 100 nm TP003 in the GoC. Right, The cumulative probability distributions of τ_w_ of all individually fitted mIPSCs in GoCs (blue; *n* =408 mIPSCs) and LuCs (red; *n* = 2300 mIPSCs). ***C***, Summary of mIPSC mean frequency, peak amplitude, and τ_w_ before (GoCs, blue; LuCs, red) and after bath application of 100 nm TP003 or 100 nm zolpidem. Note the large variance in τ_w_ in the population of GoCs, and that GoCs with a slower decay show a greater prolongation upon TP003 application. **p < 0.01 (repeated-measures parametric ANOVA, Tukey’s unequal *n* HSD *post hoc* test).

The large difference in the decay time constant of mIPSCs between GoCs and LuCs indicates a different contribution of GABAergic and glycinergic components to the IPSCs, a different subunit composition of the postsynaptic receptors, or differences in dendritic filtering of the synaptic currents. To test the first possibility, we applied the selective GABA_A_R antagonist SR95531 (20 μm) and observed a complete block of mIPSCs in both GoCs and LuCs (Fig. 2*A*), indicating that the presynaptic inhibitory cells either release only GABA that activates postsynaptic GABA_A_Rs or, if they corelease GABA and glycine, then glycine does not activate postsynaptic glycine receptors in these cells at this time point of development. To investigate potential differences in the subunit composition of postsynaptic GABA_A_Rs in the two cell types, we analyzed the pharmacological properties of mIPSCs in GoCs and LuCs (Fig. 2*B*,*C*).

We found that 100 nm zolpidem, a selective positive allosteric modulator for α1 subunit-containing GABA_A_Rs at this concentration, enhanced the mIPSC τ_w_ selectively in LuCs (from 6.0 ± 0.7 to 8.5 ± 1.4 ms; *n* = 4; *p* = 0.0039, rm-ANOVA then Tukey’s unequal *n* test), whereas 100 nm TP003, a positive allosteric modulator specific for α3 subunit-containing GABA_A_R, prolonged the mIPSC τ_w_ selectively in GoCs (from 18.1 ± 6.1 to 21.4 ± 8.5 ms, *n* = 8; *p* = 0.0091, rm-ANOVA then Tukey’s unequal *n* test). There was no significant effect of zolpidem or TP003 on the frequency or amplitude of mIPSCs in either cell type (Fig. 2*C*). The fast decay times of mIPSCs in LuCs is also consistent with postsynaptic α1β2γ2 subunit-containing GABA_A_Rs, whereas the fourfold slower decay time of mIPSCs in GoCs is consistent with α3 subunit-containing receptors (Eyre et al., 2012). We also fitted exponentials to the decay of individual mIPSCs recorded from GoCs and LuCs, and found that the distributions differed significantly (*p* < 0.0001, Mann–Whitney *U* test) between the two cell types (Fig. 2*B*). In addition, the mean 10–90% rise times of mIPSCs in LuCs (0.6 ± 0.1 ms) and GoCs (1.2 ± 0.6 ms) were comparable to those recorded from neuronal populations expressing either only α1 or only α3 as α subunits, respectively (Eyre et al., 2012), suggesting the lack of a major effect of dendritic filtering on the differences in τ_w_ between these two cell types.

**Figure 3.**
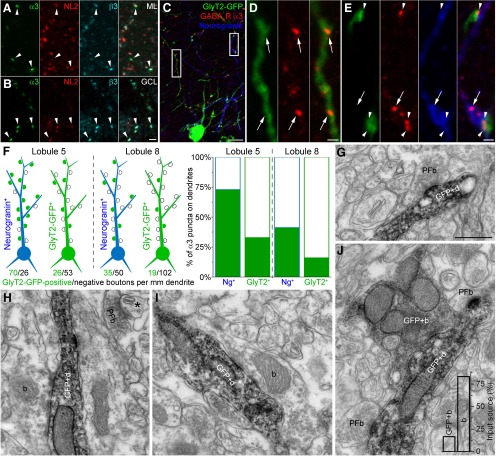
GlyT2-GFP-expressing GoC dendrites in the ML are sparsely innervated by GlyT2-GFP-expressing axons. ***A***, ***B***, Immunofluorescent labeling for the GABA_A_R α3 subunit (α3, green) in the ML (***A***) and GCL (***B***) of WT mice is sparse, but overlaps with neuroligin-2-immunopositive (NL2; red) and β3 subunit-immunopositive (β3; cyan) puncta, indicating their synaptic enrichment. Maximum intensity projections of two (***A***) or four (***B***) confocal images at 1 μm separation. Scale bars: ***A***, ***B***, 2 µm. ***C***, Immunofluorescent labeling for the GABA_A_R α3 subunit (red) in the ML of GlyT2-GFP mice is evident as multiple puncta, many of which are associated with GlyT2-GFP-expressing (green) or neurogranin-immunoreactive (blue) dendrites. Scale bar, 10 µm. ***D***, Maximum intensity projection (five confocal sections at 1 μm separation) of the left-hand boxed region in ***C*** at a higher magnification showing α3 subunit-immunoreactive puncta (arrows) associated with a GlyT2-GFP-expressing dendrite, but lacking a presynaptic GlyT2-GFP-expressing bouton. Scale bar, 1 µm. ***E***, Maximum intensity projection (five confocal sections at 1 μm separation) of the right-hand boxed region in ***C*** at a higher magnification showing intersections between GlyT2-GFP-expressing axons (arrowheads) and a neurogranin-immunoreactive dendrite. Note the presence of additional GABA_A_R α3 subunit-immunoreactive puncta not associated with a presynaptic GlyT2-GFP-expressing bouton (arrow). Scale bar, 1 µm. ***F***, Schematic proportional representation of α3 subunit-immunoreactive puncta present on GoCs in lobule 5 (left) and lobule 8 (center), categorized as expressing neurogranin (Ng^+^, blue cells) or only GlyT2-GFP (GlyT2^+^, green cells). Each punctum was categorized as either facing a presynaptic, GlyT2-GFP-expressing (green) or GlyT2-GFP negative (hollow) axonal bouton, and total puncta density per millimeter GoC dendrite for each GoC type is indicated below each cell. The graph (right) shows the percentage of GlyT2-GFP-positive (green) and -negative (hollow) boutons contacting α3-positive clusters for each type of GoC in the two lobules. ***G***, Electron micrograph showing an asymmetric synapse made by a parallel fiber bouton (PFb) onto a GlyT2-GFP-DAB-labeled dendrite (GFP+d). Scale bar: ***G*** (for ***G–J***), 500 nm***. H***, ***I***, Electron micrographs showing symmetrical synapses formed by unlabeled boutons (b) onto GlyT2-GFP-DAB-labeled dendrites. ***J***, Electron micrograph showing a symmetrical synapse formed by a GlyT2-GFP-DAB-labeled bouton (GFP+b) onto a GFP+d. Two PFbs forming asymmetric synapses onto the same dendrite are also visible. Bar graph shows the percentage of symmetrical synapses formed onto GFP+d by GFP-positive (GFP+b) and GFP-negative (b) boutons.

Given the apparently selective expression of the GABA_A_R α3 subunit by GoCs, we performed immunofluorescent labeling to investigate GABAergic synapses on GoCs. Immunofluorescent experiments following low pH-mediated antigen retrieval indicated that the α3 subunit was present in the cerebellar cortex as intensely fluorescent clusters. These clusters were much less frequent than those labeled for neuroligin-2, or the GABA_A_R α1, β2, β3, or γ2 subunits. Triple-labeling experiments revealed that the α3 subunit-immunopositive clusters were also immunoreactive for the GABA_A_R β3 subunit, the usual β subunit partner of the α3 subunit, and for neuroligin-2, indicating that these clusters correspond to GABAergic synapses in both the ML (Fig. 3*A*) and the GCL (Fig. 3*B*). Many of the neuroligin-2 puncta were not labeled for the α3 or β3 subunits, representing the much more abundant inhibitory synapses on Purkinje cell dendrites and on MLIs that express mainly the α1 and β2 subunits. Next, we performed immunofluorescent labeling for the α3 subunit in GlyT2-GFP mice and colocalized this with neurogranin, a marker selective for a subpopulation of GoCs. Immunofluorescent reactions for the α3 subunit revealed punctate labeling of the GCL and ML in GlyT2-GFP mice, similar to WT mice and rats, and demonstrated that these clusters were associated with both GlyT2-GFP-expressing and neurogranin-immunopositive dendrites (Fig. 3*C–F*). Quantification of the α3 subunit-immunopositive puncta in the ML of lobule 8 in GlyT2-GFP mice revealed that 36.0% of all α3-positive puncta were associated with dendrites expressing GlyT2-GFP (0.48 × 10^6^ puncta/mm^3^), but only 15.8% of these puncta had an adjacent presynaptic GlyT2-GFP-expressing axon-like structure (Fig. 3*C–F*). In contrast, quantification indicated that 11.0% of all α3-positive puncta were associated with dendrites immunoreactive for neurogranin. In these dendrites, 41.2% of the α3-positive puncta were associated with presynaptic GlyT2-GFP-expressing axonal structures (Fig. 3*C–F*), demonstrating that axons synapsing onto neurogranin-labeled GoCs are more likely to express GlyT2-GFP. This difference was also evident when we quantified the labeling in lobule 5: 19.0% of all observed α3 puncta were associated with dendrites expressing GlyT2-GFP, of which 32.5% had an adjacent presynaptic GlyT2-GFP-expressing axon-like structure, whereas 11.2% of all α3 puncta were associated with dendrites immunoreactive for just neurogranin, of which 71.1% had an adjacent presynaptic GlyT2-GFP-expressing axon-like structure. In addition, 10.6% of all observed α3 puncta were associated with dendrites expressing both GlyT2-GFP and neurogranin, of which 72.3% had an adjacent presynaptic GlyT2-GFP-expressing axon-like structure.

We observed that, in lobule 8, 32.3% of all α3 puncta were associated with GlyT2-GFP-expressing structures that could not be unambiguously categorized as dendrites, and the remaining 20.7% of puncta were not associated with either GlyT2-GFP-expressing or neurogranin-immunoreactive structures. These values were similar in lobule 5; 49.3% of all observed α3-positive puncta were associated with ambiguous GlyT2-GFP-expressing profiles, and 9.9% were not associated with either GoC marker. This is likely to be the consequence of incomplete labeling of GoC dendrites with neurogranin or GFP due to the extremely mild chemical fixation required for the selective synaptic labeling of the α3 subunit.

We also reconstructed the dendrites of the labeled GoCs and calculated the density of α3-positive puncta associated with a GlyT2-GFP-expressing axon-like structure or not. In lobule 8, for GlyT2-GFP-expressing dendrites, there were 19 and 102 α3-positive puncta/mm GoC dendrite with and without GlyT2-GFP-expressing inputs, respectively. For neurogranin-immunoreactive dendrites, there were 35 and 50 α3-positive puncta/mm dendrite with and without GlyT2-GFP-expressing inputs, respectively. In lobule 5, for GlyT2-GFP-expressing dendrites, there were 26 and 53 α3-positive puncta/mm dendrite with or without GlyT2-GFP-expressing inputs, respectively. Finally, for neurogranin-immunoreactive dendrites in lobule 5, there were 70 and 26 α3-positive puncta/mm dendrite with and without GlyT2-GFP inputs, respectively (Fig. 3*F*). These data indicate that although there may be subtle regional differences within the cerebellar cortex, the majority of GABA_A_R α3 subunit-containing synapses on GlyT2-GFP-expressing dendrites do not have a GlyT2-GFP-expressing presynaptic partner. In contrast, the proportion of GlyT2-GFP-expressing inputs to neurogranin-immunoreactive GoCs is substantially higher, which is consistent with the finding of a recent report (Ankri et al., 2015) demonstrating that these cells receive a selective GlyT2-expressing input from deep cerebellar nucleus (DCN) neurons.

Next, we performed another set of experiments for estimating the proportion of GlyT2-GFP-expressing inputs to GlyT2-GFP-expressing GoC dendrites. We used an immunoperoxidase reaction for GFP and processed the sections for EM (GlyT2-GFP-DAB). We systematically sampled the ML in lobule 8 of GlyT2-GFP-DAB reactions and analyzed 117 dendritic profiles at the ultrastructural level. The vast majority of synaptic contacts onto GlyT2-GFP-DAB dendrites was asymmetric and characteristic of glutamatergic parallel fiber synapses (546 of 570 boutons, 95.8%; 553 of 578 synapses, 95.7%; Fig. 3*G*). Of the 24 boutons forming symmetrical synapses onto GlyT2-GFP-DAB dendrites, 20 boutons were not labeled for GlyT2-GFP-DAB (Fig. 3*H*,*I*). Only four appositions (16.6%) between GFP-expressing axons and GFP-expressing dendrites were observed; three formed synapses with one active zone (AZ) each, and one formed a synaptic contact with two AZs on the target dendrite (Fig. 3*J*).

**Figure 4.**
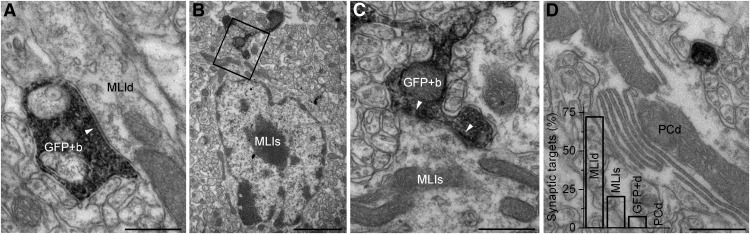
GlyT2-GFP-expressing axons target MLIs. ***A***, Electron micrograph showing a GlyT2-GFP-DAB-positive axon varicosity (GFP+b) making a symmetrical synapse (arrowhead) with a smooth, thin, nonspiny MLI dendrite (MLId). Scale bar, 500 nm. ***B***, Electron micrograph showing a GFP+b establishing a symmetrical synapse with a MLI soma (MLIs). Scale bar, 2 μm. ***C***, Electron micrograph of the boxed region in ***B*** showing the synaptic junctions (arrowheads) at a higher magnification. Scale bar, 500 nm. ***D***, Electron micrograph showing a thin GlyT2-GFP-DAB-immunoreactive interbouton axon segment (black precipitate) adjacent to, but lacking a synapse with, a PC dendrite (PCd). Scale bar, 500 nm. Bar graph shows the percentage of synapses formed by GlyT2-GFP-DAB-immunoreactive axons in MLId, MLIs, GlyT2-GFP-DAB-immunoreactive dendrites (GFP+d), and PCd.

We also observed and examined a total of 54 GlyT2-GFP-DAB boutons, of which 39 (72.2%) formed 44 AZs on MLI dendrites, and 11 (20.4%) established 18 AZs on MLI somata (Fig. 4*A–C*); the remaining four boutons contacted GlyT2-GFP-DAB dendrites, as detailed in the previous paragraph. We never observed synapses on Purkinje cell dendrites or spines, even though the GlyT2-GFP-DAB axons were sometimes observed to come into direct contact with these structures (Fig. 4*D*). We also observed six instances of contacts between GlyT2-GFP-DAB-labeled dendrites, three of which had puncta adherentia-like membrane specializations and potential electrical synapses, although these were difficult to unequivocally identify in our material. In summary, these data corroborate our light microscopy (LM) observations, indicating that the majority of inhibitory inputs to GlyT2-GFP-expressing GoCs in the ML do not originate from GlyT2-GFP-expressing axons.

**Figure 5.**
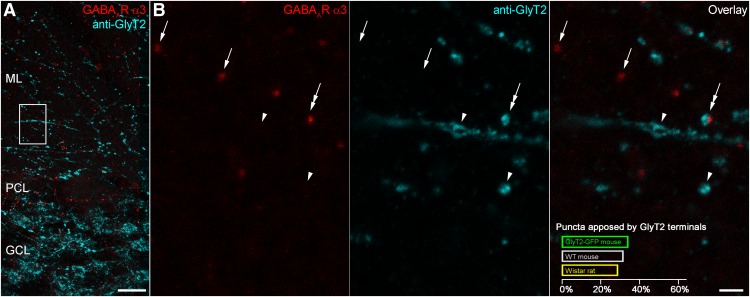
Only one third of GABA_A_R α3 subunit-immunoreactive puncta face varicosities immunolabeled for GlyT2. ***A***, Maximum intensity projection (six confocal sections at 1 μm separation) from a Wistar rat section immunolabeled for GABA_A_R α3 (red) and GlyT2 (cyan). Scale bar, 20 µm. ***B***, Enlarged view of the boxed region in ***A*** showing GABA_A_R α3-immunoreactive puncta (left, red) either not associated with (arrows) or closely associated with (double-headed arrow) GlyT2-immunoreactive terminals (middle, cyan), as can be seen in the overlay (right). Quantification of GABA_A_R α3-immunoreactive puncta is shown for three genotypes in the lower part of the overlay panel. All images are maximum intensity projections of four confocal sections at 1 μm separation. Scale bar, 2 µm.

The GlyT2-GFP mouse line is subject to mosaic expression of the transgene, resulting in a variable degree of GFP expression in glycinergic cells (Husson et al., 2014). In order to test whether the low proportion of α3-positive puncta facing GlyT2-GFP-expressing terminals is the consequence of an incomplete expression of the GFP in glycinergic cells, we used an immunofluorescent reaction for GlyT2 to label glycinergic terminals, irrespective of their GFP content in the cerebellar cortex of rats and mice, and combined this with immunofluorescent labeling for the α3 subunit (Fig. 5). Quantification of the immunoreaction indicated that only a minority of α3 subunit-immunoreactive puncta was apposed by GlyT2-immunoreactive varicosities (28.5% in Wistar rat; 31.3% in WT mouse; 33.8% in GlyT2-GFP mouse; Fig. 5*B*). Conversely, approximately half or less of all GlyT2-immunoreactive terminals were facing α3 subunit-immunoreactive puncta (27.0% in Wistar rat; 40.7% in WT mouse; 55.4% in GlyT2-GFP mouse). These results add further evidence for a lack of glycine/GlyT2 in the majority of inhibitory inputs to GoC dendrites located in the ML.

**Figure 6.**
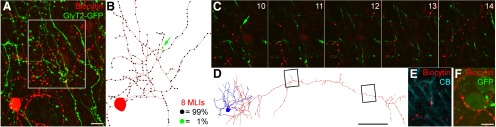
GlyT2-GFP-expressing dendrites receive negligible inputs from MLI axons. ***A***, Maximum intensity projection image of a confocal image *Z*-stack of an *in vitro* recorded and biocytin (red)-labeled MLI with axons spreading among GlyT2-GFP-expressing processes (green). Scale bar: ***A*** (for ***A–C***), 10 μm. ***B***, Neurolucida reconstruction of the soma and axon of the recorded cell (red; black circles indicate varicosities; green circles indicate varicosities next to GlyT2-GFP-expressing dendrites). The number of reconstructed cells and the percentage occurrence of each varicosity type are indicated. ***C***, Boxed area in ***A*** shown as a series of confocal image planes highlighting a single putative contact (green arrow). Note that most varicosities of the biocytin-filled axon have unlabeled targets, despite numerous GlyT2-GFP-expressing structures in the vicinity. ***D***, Neurolucida reconstruction of a different MLI (soma and dendrites in blue, axon in red). Scale bar, 50 μm. Boxed regions correspond to the fluorescent images shown in ***E*** (left) and ***F*** (right). ***E***, The axon of the MLI in ***D*** makes a bouton in apposition (red) to a calbindin-immunoreactive (CB; cyan) Purkinje cell dendrite. ***F***, The same axon also has several boutons in close apposition with a weakly GlyT2-GFP-labeled MLI soma (GFP; green). Scale bar: ***F*** (for ***E***, ***F***), 5 μm.

As neither Purkinje nor GoC axons arborize in the ML, the most obvious cells giving rise to GlyT2-GFP-negative GABAergic axons that could, in principle, innervate GoC dendrites in the ML are MLIs. Because the existence of MLI-to-GoC connections is debated, we investigated whether this connectivity could explain the high occurrence of GlyT2-GFP unlabeled synapses on GoC dendrites by recording and intracellularly filling MLIs in acute slices (Fig. 6). From the eight cells that were recovered with sufficiently intact axonal arbors, we reconstructed a total of 7909 μm of axon with 1870 boutons, but observed that only 19 boutons (1.02%) were closely opposed to GlyT2-GFP-expressing dendrites (Fig. 6*C*). In contrast, appositions were frequently observed with Purkinje cell dendrites (labeled for calbindin; Fig. 6*E*) and MLI somata (which transiently express the GlyT2-GFP transgene during their development and are thus weakly labeled in our sample; Fig. 6*F*). Our data argue against the presence of profound MLI-to-GoC chemical synaptic connectivity and are consistent with either extremely weak or a lack of innervation (Hull and Regehr, 2012).

However, 1% of an abundant bouton population could still be a significant source of input to an infrequent cell population. From our reconstructions, we quantified that, on average, MLIs have 234 ± 37 boutons/cell, which is similar to previous estimates by Sultan and Bower (1998) using Golgi impregnation. To estimate the proportion of GABAergic inputs to GoCs that might originate from these MLIs, we compared the density of MLIs (48,000/mm^3^, measured in the present study) and GoCs (∼4700/mm^3^ GlyT2-positive GoCs, as reported by Simat et al., 2007; Billings et al., 2014), giving a ratio of 10:1 (see also Korbo et al., 1993). This implies that a maximum of 23 GABAergic synapses (234 boutons/cell × 1.02% contacting GoCs × 10:1 cell ratio) could originate from MLIs onto each GlyT2-expressing GoC, which would constitute a maximum of 14% of all GABAergic synapses (163) on GoC dendrites in the ML (1350 ± 545 μm of dendrite/GoC × 0.121 GABA_A_R α3 puncta/μm GoC dendrite).

## Discussion

With the aid of GlyT2-GFP animals, patch-clamp recordings, and *post hoc* morphological analysis, we were able to identify GoCs and LuCs, and to investigate their place in the GABAergic circuit of the cerebellar cortex. By identifying the GABA_A_R α3 subunit as a selective marker for inhibitory synapses on GoC dendrites, we provided LM evidence that GABAergic innervation of GoCs by local inhibitory axon populations is sparse. GlyT2-GFP-expressing axons provide a maximum of 16% of all GABAergic synapses on GlyT2-GFP-expressing GoC dendrites in the ML, with potential origin from LuCs, globular cells, or DCN cells. Glycine-immunoreactive axons not expressing GlyT2-GFP could account for a further 18% of these inputs. Our data argue against the presence of MLI-to-GoC connections, but we cannot exclude the possibility of a sparse innervation (a maximum of 14%). However, even if this connection exists, more than half of the inhibitory inputs to GoCs must arise from purely GABAergic sources that are not located within the cerebellar cortex.

### Local inhibitory inputs to GoCs

Golgi cell dendrites occupy all layers of the cerebellar cortex, and both basal and apical dendrites contain GABA_A_R α3 subunit-positive puncta, demonstrating that these cells receive inhibitory inputs in all layers. Thus, all inhibitory neurons of the cerebellar cortex, as well as any extracortical inputs, could provide GABAergic/glycinergic innervation of GoCs. Molecular layer IN axons are confined to the ML, where they innervate each other and provide a powerful inhibition to Purkinje cells (Vincent and Marty, 1996; Kondo and Marty, 1998). It has been assumed that these axon terminals are also responsible for the inhibitory inputs to GoC dendrites in the ML (Palay and Chan-Palay, 1974). Here we investigated the potential inhibitory connections between MLIs and GoCs using intracellularly filled MLIs and *post hoc* LM analysis of the spatial relationships between their axon terminals and GlyT2-GFP-expressing GoC dendrites. Our results demonstrate that a maximum of 1% of the MLI boutons are in close apposition with GlyT2-GFP-expressing GoC dendrites, which could in principle provide direct synaptic inputs. However, due to the insufficient ultrastructural preservation in our mildly fixed tissue, we could not verify these appositions as synaptic connections using EM. Even if they were all synapses, and even though MLIs outnumber GoCs by 10:1, we calculate that a maximum of 14% of all GABAergic inputs to GoCs could arise from MLIs in the ML. These results are in agreement with those of Hull and Regehr (2012), who used optogenetic stimulation techniques and paired recordings to demonstrate the lack of functional connectivity between MLIs and GoCs.

Lugaro and globular cells are the least studied inhibitory INs of the GCL. Their classifying feature is that they have a minimal axonal arbor in the GCL, but instead provide a long-range innervation in the ML. As demonstrated here and in previous studies (Zeilhofer et al., 2005; Simat et al., 2007), LuCs and globular cells express GFP in these GlyT2-GFP transgenic animals and are likely to be responsible for at least some fraction of the ∼16% GABA/glycinergic inputs of GlyT2-GFP-expressing GoCs in the ML. Although LuC/globular cell input to GoCs is sparse, they seem to be sufficient to generate large-amplitude rhythmic IPSCs upon 5-HT application (Dieudonné and Dumoulin, 2000; Hirono et al., 2012). Our EM analysis demonstrated that the GlyT2-GFP-expressing axon terminals exclusively innervate INs (MLIs and GoCs), but avoid the innervation of the principal (Purkinje) cells of the cerebellar cortex. GoCs innervate glutamatergic GrCs, and MLIs clearly innervate Purkinje cells; therefore, globular cells and LuCs are the sole IN-selective INs of the cerebellar cortex. Such IN-selective INs have been described in many brain regions, including the hippocampus (Acsády et al., 1996; Gulyás et al., 1996), the neocortex (Meskenaite, 1997; Melzer et al., 2012), and the main olfactory bulb (Eyre et al., 2008), and our data indicate that LuC/globular cells are also included in this category.

Inhibitory inputs to GoCs could also arise from Purkinje cell local collaterals arborizing in the superficial GCL and PCL. Calbindin-immunoreactive Purkinje cell local axon collateral boutons have been shown in close apposition to LuCs and globular cells adjacent to the PCL (Lainé and Axelrad, 2002; Simat et al., 2007). Purkinje cells are spontaneously active in *in vitro* slices (Häusser and Clark, 1997), strongly suggesting causality between their cessation of activity and the significant reduction in LuC IPSC frequency that we observed after the application of TTX. Consistent with this, globular cells have been shown to receive monosynaptic inhibitory inputs from Purkinje cells (Hirono et al., 2012). In contrast, we observed a very low frequency of sIPSCs in GoCs and no significant change in the frequency following TTX application, indicating that a significant proportion of the GABAergic inputs of GoCs are unlikely to be provided by Purkinje cells, but must instead arise from sources that are either spontaneously inactive or are not present in our acute slices. As stellate and basket cell networks are also spontaneously active in slices (Häusser and Clark, 1997), this gives further support to the idea that MLIs are also unlikely to provide major inhibitory inputs to GoCs.

Factors governing the occurrence of spontaneous activity in GoCs are also not well defined, although the age of the animals and the recording temperature are likely to be critical (Dieudonne, 1998; Forti et al., 2006). We found that spontaneously active GoCs are much more frequently encountered in rat versus mouse slices (Rudolph et al., 2015). Although we cannot entirely rule out the possibility that mouse GoCs are more vulnerable to the slicing procedure, our GoCs in mouse slices appeared healthy; success rates of patching, access resistances, and firing patterns were similar to those obtained from rats. The fact that the rheobase current is smaller and the RMP is more depolarized in rat GoCs suggests that they are more excitable than mouse GoCs, although the maximum firing rates upon current injection were similar. We suggest that the difference might lie in distinct ion channel expression patterns between rat and mouse GoCs.

Golgi cells have extensive axonal arbors in the GCL and provide phasic and tonic inhibition to GrCs. The issue of whether GoCs provide chemical GABAergic innervation to each other has been debated; two recent studies using paired recordings found no evidence for this connection (Dugué et al., 2009; Vervaeke et al., 2010), but Hull and Regehr (2012) demonstrated a GoC-to-GoC connection probability of ∼20%. A potential reason for this discrepancy might be the consequence of tonic presynaptic GABA_B_ receptor-mediated inhibition of GABA release from GoC axons. Dugué et al. (2009) and Vervaeke et al. (2010) did not eliminate GABA_B_ receptor-mediated presynaptic inhibition, whereas Hull and Regehr (2012) performed their experiments in the presence of a GABA_B_ receptor antagonist. We did not inhibit GABA_B_ receptors in our recordings, and thus GoC-to-GoC synaptic inhibition is unlikely to have contributed to our functional measurements. Anatomically, even if GoCs provide GABAergic innervation to each other, these inputs are confined to the GCL and cannot be responsible for the >50% “missing” inputs on GoC dendrites in the ML. Due to the high density of GlyT2-GFP-expressing axonal and dendritic processes in the GCL, we were unable to discern the extent to which GlyT2-GFP-expressing presynaptic axons are in direct apposition with GlyT2-GFP-expressing GoC basal dendrites at α3-positive puncta. However, we consider it possible that this coverage is similar to that of the apical dendrites, as is suggested by the similar density of GABA_A_R α3 puncta in these two regions (rat ML, 0.002 ± 0.001 puncta/µm^3^; GCL, 0.002 ± 0.0003 puncta/µm^3^).

### Extracortical inhibitory inputs to GoCs

Recent work by Ankri et al. (2015) elegantly demonstrated that a population of GlyT2- and GAD65-expressing cells in the DCN projects to the cerebellar cortex and targets a subgroup of GoCs that are spontaneously active and express neurogranin, but not GlyT2. In agreement with this report, here we show that 40–70% of α3-immunoreactive synapses on neurogranin-expressing, GlyT2-GFP-negative dendrites were contacted by GlyT2-GFP-expressing axons. This proportion is higher than that found on GlyT2-GFP-expressing GoCs (16–34%). A plausible explanation of these data is that GlyT2-GFP-expressing GoCs receive most, if not all, of their GlyT2-GFP-expressing inputs from local LuCs/globular cells, whereas the neurogranin-positive, GlyT2-GFP-negative GoCs receive some of their inputs from LuCs/globular cells and an additional major input from GlyT2-GFP-expressing DCN cells. Because only 40% of DCN glycinergic cells express GFP in GlyT2-GFP mice (Husson et al., 2014), it is possible that the lack of GFP in the majority of the boutons contacting α3-positive puncta in GoC dendrites (both GlyT2-GFP-expressing and neurogranin-immunopositive populations) could originate from these glycinergic, but GFP-negative, DCN neurons. Our results using immunolocalization with an anti-GlyT2 antibody in WT mice and Wistar rats, however, argues against this possibility.

Ankri et al. (2015) demonstrated that the majority of GAD65-expressing DCN cell axons in the cerebellar cortex also express GlyT2, but did not test the expression of GAD67, which is also known to label DCN neurons (Uusisaari et al., 2007). Although DCNs have not been extensively characterized (Uusisaari and De Schutter, 2011), a report (Bäurle and Grüsser-Cornehls, 1997) indicated that only 23.7% of all DCN cells immunoreactive for GABA also expressed GlyT2. Many small GABAergic cells in the DCN project to the inferior olive (Batini et al., 1989), but this does not rule out the possibility that they also project to the cerebellar cortex, or even that all purely GABAergic DCN neurons are of this type. We therefore predict that a proportion of DCN GABAergic INs directly influences the cerebellar cortex inhibitory networks (Uusisaari and De Schutter, 2011). In summary, our results indicate that the majority of the inhibitory inputs to GlyT2-GFP-expressing GoCs in the ML must arise from neurons outside the cerebellar cortex that contain GABA, but not glycine.

## Synthesis

The decision was a result of the Reviewing Editor Alfonso Represa and the peer reviewers coming together and discussing their recommendations until a consensus was reached. A fact-based synthesis statement explaining their decision and outlining what is needed to prepare a revision is listed below. The following reviewer(s) agreed to reveal their identity: Jean-Marc Fritschy

Dear authors,

Your ms. has been reviewed by two referees that found your data interesting for the community. There are not that much concerns about experiments, data and interpretations and the quality of the research is positively appreciated. However both referees find that your statement and tittle are too strong and that it would be required a "more direct evidence for a GABAergic projection from the deep cerebellar nuclei (or elsewhere) to the cerebellar cortex "before making such a strong statement. It is highlighted the difficulty on providing definite conclusions (Extra-cerebellar cortical origin of the majority of inhibitory inputs to Golgi cells) based only on negative results (that small minority of the alpha3-containing synapses on mouse glycinergic Golgi cell dendrites in the molecular layer receive a glycinergic axon"). I'm therefore suggesting you to modify you title and conclusions accordingly to the comments of the referees. No new experiments are required in principle.

I'm also pasting below more specific comments of referees that need to be considered in your revision.

Page 11 / Figure 1:

"Quantitative comparisons indicated that current rheobase was significantly larger (98.0 {plus minus} 58.0 pA vs. 30.0 {plus minus} 21.2 pA; p = 0.0002, Mann-Whitney U test) and input resistance was significantly lower (207.0 {plus minus} 89.8 Mohms vs. 556.3 {plus minus} 239.5 Mohms; p = 0.0001, Mann-Whitney U test; Fig. 1C) in GoCs versus LuCs. " - as low input resistance directly results in larger rheobase, it would be useful to know the rheobase normalised for input resistance or capacitance of the cells.

Were the data corrected for junction potential?

The example traces shown in Fig. 1 (especially the LuC) aren't very good and look as if the cells weren't healthy, raising some concern over the measurements provided. Were the current-clamp experiments also recorded using high-Cl internal solution?

P. 12 / Figure 2:

with the intracellular solutions used, what would be the estimated chloride reversal potential?

As it is already known that the glycine-mediated synaptic responses are much smaller than the GABA-mediated, the pharmacological experiments aiming at uncovering the glycinergic component in mIPSCs should be done in opposite order - block glycine receptors first and see how much is remaining. Especially with the surprisingly low amplitude of IPSCs and signal-to-noise-ratio in GoCs, residual glycinergic component might be invisible.

the example LuC trace shown in Figure 2 has large IPSCs that do not seem to fit what is given in the graph on the right (mean amplitude 20 pA)

The small increase in the mIPSC decay with TP003 in GoCs is taken to show that the GABA-receptors in GoCs all contain the alpha3-subunit. As it is also possible that the GoCs contain a mixture of receptors with different compositions, in addition to average values, comparisons must be done on distributions of the measurements (histograms or cumulative plots).

The authors spend considerable time wondering about why their GoCs are mostly silent. An easy explanation for this is the known fact that the GlyT2+ GoCs (that they authors target) are not spontaneously active, unlike the GABAergic GoCs (see Dugue et al, 2009 and Ankri &amp; al, 2015). The GlyT2+ GoCs also are the largest population of GoCs, and therefore they are the most likely patched cells unless a GABA-specific label is used. Thus, the section related to experiments in rats can be removed.

P. 14:

"The large difference in the decay time constant of mIPSCs between GoCs and LuCs indicates either a different contribution of GABAergic and glycinergic components to the IPSCs or a different subunit composition of the postsynaptic receptors. " - different decay time constant of the IPSCs can be also due to different location on the dendrites (distal vs proximal, differences in dendritic active properties ...) comparing rise times should be helpful.

Please show the data for co-localization of alpha3 with gamma2 and beta3; as the conclusions of the work to quite some extent depend on the presumption that all of the inhibitory synapses in GoCs have alpha3, this issue demands proper presentation.

P. 15:

The section explaining distributions of immunolabeled structures is impossible to understand without a clarifying schematic. For instance: "Quantification indicated that 11.0% of all GABAAR 3 subunit puncta were associated with neurogranin-immunoreactive dendrites. " - does this mean neurogranin-positive and GlyT2-negative?

"We observed that 32.3% of all puncta were associated with GlyT2-GFP-expressing structures that could not be unambiguously categorized as dendrites, and the remaining 20.7% of the puncta were not associated with either GlyT2-GFP- or neurogranin- immunoreactive structures. This is likely to be the consequence of incomplete labelling of GoC dendrites with neurogranin or GFP due to the extremely mild chemical fixation required for the synaptic labelling of the alpha3 subunit. " - another logical possibility is that they would be on MLI or PN dendrites.

The section regarding lobule-dependent expression is vague; at least, show the results in a figure from the examined tissue.

Specific comments (minor):

P. 4: "Lugaro cells are GCL INs with horizontal dendritic arbors running parallel with the Purkinje cell layer (PCL) and axons arborizing in the ML. " - it is well-known that the LuC axons also branch and make synapses in the GrCL (Laine &amp; Axelrad 1996, 2002 as well as Melik-Musyan &amp; Fanardzhyan 2004)

P. 5, line 3-4: "we monitored all cells" - should be, monitored neuronal activity in cell-attached mode or such

P. 6: give details on how AP parameters were defined and measured

P.8, methods: details missing on other GABAaR subunit staining

P.9: sex of mice used for EM missing

P11 / Figure 2: color coding is confusing - please use same colouring scheme for GoC and LuC throughout the figure panels

P. 16:

"We also examined a total of 54 GlyT2-GFP-expressing boutons, out of which 39 (72.2%) formed 44 AZs onto MLI dendrites " - how do you know they are MLI dendrites?

P17:

- "In contrast, appositions were frequently observed with Purkinje cell dendrites (labeled for calbindin; Fig. 6E) and MLI somata (Fig. 6F). " - how are MLIs labeled?

P.22:

- "However, we consider it possible that this coverage is similar to that of the apical dendrites, as is qualitatively suggested by the density of GABAAR alpha3 puncta in these two regions (Fig. 5). " - I can not draw any conclusions like that from Figure 5; in 5A, the red dots are too weakly visible and if anything, I'd say there are much less of them in the GrCL than in ML.

P. 23:

- "Tracing studies have indicated that the major routes of information flow to the cerebellar cortex all synapse within the DCN, which thus form the gateway to the cerebellum (Ruigrok, 2011). " This is a rather strange view; all inputs into cerebellum do synapse within the DCN but via collaterals, the main branch of which continues to the cerebellar cortex. I can not see how DCN could be described as the input gateway (even though it is the output gateway for sure).

In general, the MS would benefit from careful rewrite as it is in many places imprecise in wording; e.g., it is not always clear if they are writing about mIPSCs or sIPSCs, or which immunolabel product is being described.
